# Establishing a Scientific Consensus on the Cognitive Benefits of Physical Activity

**DOI:** 10.3390/ijerph17010029

**Published:** 2019-12-18

**Authors:** Nesrin Nazlieva, Myrto-Foteini Mavilidi, Martine Baars, Fred Paas

**Affiliations:** 1 Department of Psychology, Education and Child Studies, Erasmus University Rotterdam, 3062 PA Rotterdam, The Netherlands; NesrinNazlieva@outlook.com (N.N.); baars@essb.eur.nl (M.B.); 2 Priority Research Centre for Physical Activity and Nutrition, School of Education, University of Newcastle, Newcastle, Callaghan NSW 2308, Australia; Myrto.Mavilidi@newcastle.edu.au; 3 School of Education/Early Start, University of Wollongong, Wollongong NSW 2522, Australia

**Keywords:** scientific consensus, physical activity, physical fitness, cognition, cognitive function, learning

## Abstract

Research suggests that physical activity can be used as an intervention to increase cognitive function. Yet, there are competing views on the cognitive effects of physical activity and it is not clear what level of consensus exists among researchers in the field. The purpose of this study was two-fold: Firstly, to quantify the scientific consensus by focusing on the relationship between physical activity and cognitive function. Secondly, to investigate if there is a gap between the public’s and scientists’ interpretations of scientific texts on this topic. A two-phase study was performed by including 75 scientists in the first phase and 15 non-scientists in the second phase. Participants were asked to categorize article abstracts in terms of endorsement of the effect of physical activity on cognitive function. Results indicated that there was a 76.1% consensus that physical activity has positive cognitive effects. There was a consistent association between scientists’ and non-scientists’ categorizations, suggesting that both groups perceived abstracts in a similar fashion. Taken together, this study provides the first analysis of its kind to evaluate the level of consensus in almost two decades of research. The present data can be used to inform further research and practice.

## 1. Introduction

Over the past decade, the popular, commercial, and scientific interest in physical exercise has grown. According to the International Health, Racquet and Sportsclub Association [1], the health club industry revenue was estimated at $87.2 billion in 2017 [1]. Over 201,000 clubs served 174 million members around the world. In the US alone, the number of health club members in 2017 (*n* = 60.9 million) has increased by 33.6%, compared to 2008 (*n* = 45.6 million) [1]. What is more, a recent ISI Web of Science search on the term “physical exercise” (PE) revealed a 269% increase of scientific papers in 2018 (*n* = 9574), contrasted to those of a decade earlier (*n* = 3557). Mass media coverage has gone as far as to compare the health consequences of sitting to those of smoking. A recent research analysis of news articles found nearly 300 articles claiming that “sitting is the new smoking” [2]. Such claims, however, were found to be inaccurate since “absolute risk differences for smoking far outweigh those for sitting, except for type 2 diabetes” [3]. Additionally, the fact that physical exercise has gained much attention over the past decade has also led to various studies regarding exercise’s effects on the human body. Interestingly, next to positive effects on health, physical exercise can also affect the human brain and cognition. However, there are competing views on the cognitive effects of physical activity and it is not clear what level of consensus exists among researchers in the field. In this study we tried to quantify the scientific consensus on the relationship between physical activity (PA) and cognitive function. In addition, we investigated if there is a gap between the public’s and scientists’ interpretations of scientific texts on this topic. We start by defining the concept of physical activity and provide scientific evidence on its effect on. Subsequently, we discuss the competing views on the effects of physical activity and its potential physiological and the psychological mechanisms.

To begin with, according to the World Health Organization (2010) [4], physical activity is “any bodily movement produced by skeletal muscles that requires energy expenditure.” As such, PA includes any motor behavior in daily and leisure activities. Housework activities classify as a form of PA, and as such, it has been shown that they may have a greater beneficial effect on executive function compared to other physical activities, through the activation of the right ventrolateral prefrontal cortex (R-VLPFC) [5]. PE, however, is “a sub-classification of PA that is planned, structured, repetitive, and has as a final or an intermediate objective the improvement or maintenance of one or more components of physical fitness” [4]. Aerobic and anaerobic activity, characterized by a certain frequency, duration, and intensity, are examples of PE. Conversely, physical fitness (PF) is one’s ability to perform aspects of daily activities and sports with optimal performance, strength, and endurance [6]. Another term that is used throughout the manuscript is “cognition” which is a set of mental processes that contribute to cognitive measures such as action, perception, intellect, and memory [7].

Research has shown that PE might exert rather small benefits on cognitive capacities when compared to other enhancers such as caffeine, sugar, or modafinil; however, it has additional benefits, such as enhanced mental or physical health, without side effects [8]. According to the U.S. Department of Health and Human Services (2018) [5], PA can improve cognition and reduce the risk of depression in youth. For older adults, it can reduce symptoms of anxiety and depression, and improve cognition for those with dementia, multiple sclerosis, ADHD, and Parkinson’s disease [5]. Lack of physical activity and physical exercise has also been associated with a higher risk of dementia among older populations [9]. In school-age children, it was determined that physical exercise benefits academic achievement, perceptual skills, verbal and mathematical ability, and intelligence [10]. Physical activity in the form of moderate cycling exercise can also enhance neurocognitive processing in adolescents with intellectual and developmental disabilities [11]. Furthermore, a meta-analysis of randomized controlled trials revealed that aerobic exercise training could improve processing speed, executive function, and attention [12]. However, the effects on working memory were less consistent [12]. Mandolesi et al. (2018) [13] pointed out that both chronic and aerobic PE can achieve similar benefits and that they play a role in counteracting normal and pathological aging.

Physical activity has also been identified as a protective factor against age-related cognitive decline. This notion is supported by a neuroimaging study assessing PA in individuals in their early seventies [14]. They determined that PA preserves the structural volume in the prefrontal and temporal cortices after nine years of follow-up. Additionally, preserved grey matter volume was observed in several cortical and subcortical regions, such as the hippocampus [14]. Another study found that aerobic training can even significantly increase hippocampal volume in older women with mild cognitive impairment [15]. Physical activity has also been associated with changes in grey and white matter structures, metabolite concentration, and corticospinal/intracortical excitability [16]. These changes were observed in young, adult, and elderly populations but were reversed in athletes with concussions [16]. As in almost every other cognition-enhancing method, some people seem to benefit more than others [8].

Scientists do not yet know the precise mechanisms of the way exercise changes the structure and function of the brain. For instance, a study by Brisswalter, Collardeau, and René (2002) [17] points at an increase in arousal level related to physical exertion as a potential mechanism for improvement in cognitive performance during exercise. Although there is no clear functional hypothesis that explains the relationship between arousal and exercise, motivation and attention have been mentioned as important psychological mediators in this relationship [17].

Another mechanism was uncovered by Mata, Thompson, and Gotlib (2010) [18]. They demonstrated that a *brain-derived neurotrophic factor* (*BDNF*) genotype moderates the protective effect of PA on depressive symptoms in adolescent girls. The researchers tested 82 girls by using a psychological and biological test. They found that physical activity served as a protective factor for girls in the high-risk group who carry a *BDNF* gene variation, called met allele. On the contrary, the girls with a different, homozygous variant, called val allele, did not benefit as much from physical activity. The interaction between the physical activity and the *BDNF* gene has not manifested with respect to depression. The majority of the studies in both adolescents and adults have failed to replicate the effect when it comes to depression outcomes [19].

A literature review by Marmeleira (2012) [20] draws attention to multiple physiological and psychological mechanisms that support a positive relationship between PA and cognition, emphasizing the potential effects of different types of exercise. For instance, cardiovascular (aerobic) exercise has been considered to mediate the positive association between physical activity and cognition. This is also known as “the cardiovascular fitness hypothesis.” The cognitive benefits from aerobic exercise are thought to be due to BDNF, glucose availability, cerebral oxygen, changes in cerebral structure, and neurotransmitters’ levels, which themselves have been associated with improved cognitive performance [20,21].

Another line of research line builds on the human movement effect [22,23]. Using the theoretical frameworks of cognitive load theory and embodied cognition, Sweller and colleagues (2019) [23] provided an explanation for positive effects of fine movements on cognition and learning. It is assumed that fine movements, such as making gestures during problem solving (e.g., finger counting, pointing, tracing), or gross motor movements, such as enacting to learned words, can improve learning by sharing the load between the cognitive and motor systems (i.e., cognitive offloading) [24,25] and by providing additional cues that can be incorporated in cognitive schemas and used in subsequent knowledge retrieval [22]. Mavilidi et al. (2018) [26] have suggested that for movements to be effective for learning they need to be integrated into and relevant for the learning task.

### 1.1. Scientific Consensus

The “scientific consensus” approach in this field is relatively new, and to date there is only one study which has employed it. An expert panel approach was proposed by Singh et al. (2019) [27]. The authors conducted a systematic review with an international expert panel to evaluate the evidence on the effects of PA interventions on cognitive and academic performance in children. They determined that the current state of scientific literature is inconclusive, regarding the beneficial effects of physical activity interventions on cognitive and academic performance in children [27].

A longitudinal experiment on the potential role of exercise in preventing cognitive decline found that exercise helped play a protective role in cognitive functioning in elders over time [28]. Their subjects were rural elders, 65 years of age or older of low socioeconomic status and education. The results showed that a higher exercise level was associated with an absence of substantial cognitive decline two years later, even after adjusting for variables such as age, sex, education, previous level of cognitive function, self-rated health, and exercise frequency [28]. This could be explained by the fact that increasing energy output from a variety of physical activities is related to larger gray matter volumes in the elderly, regardless of cognitive status [21]. Another follow-up study in China found that people with limited physical activity had a higher risk of developing dementia [29].

However, other longitudinal studies failed to find such an association. For instance, a 7-year prospective study in Japan discovered that neither work nor leisure PA was protective against Alzheimer’s disease for Japanese participants (*n* = 828) [30]. Additionally, a systematic review study revealed largely insufficient evidence for the effectiveness of any exercise intervention on promoting cognitive function and preventing cognitive decline in older adults [31]. What is more, they also found that most of the trial studies were small, underpowered, and unable to assess the clinical significance of cognitive test outcomes [31].

The above-mentioned contradictions in research findings have been addressed by mass media, such as Time magazine [32] and STAT, produced by Boston Globe Media [33]. Currently, there is no complete consensus regarding the effects of PE on the human brain. When evidence is uncertain or not quantified, people tend to erroneously reach conclusions about the gravity of evidence. This is a result of a well-established human information processing mechanism, called the “availability heuristic” [34]. In such instances, a scientific consensus is a tool that serves as a form of social proof, easily comprehended by laymen and experts alike [35]. The major stakeholders in creating and communicating consensus are scientists and non-scientists. Scientists are the ones who determine and quantify the scientific consensus, which is important, as it provides a novel methodology for assessing the scientific weight of evidence. They do that by implementing structured communication techniques and/or methods such as the Delphi method [36,37]. Consensus is crucial as it safeguards the public against influential misinformation. This is where the non-scientists play a role as communicating the scientific consensus has a powerful effect on realigning public views of the issue with expert opinions [35]. For example, there have been conflicting views on the state of evidence whether brain games can improve cognitive function in daily life [38,39]. In an attempt to determine the state of evidence, Simons et al. (2016) [40] reviewed literature cited by brain-training proponents and leading companies and concluded that there was not sufficient evidence to justify the claim that brain training is an effective tool for enhancing cognition in the real world. They found that such studies lacked consistency and had methodological shortcomings, such as lack of preregistration, incomplete reporting, small sample sizes, and no controls for placebo effects [40]. These types of reviews are important because they resolve ambiguities in the current state of knowledge and explore the present state of understanding on a topic [41]. For this reason, the present study sought to clarify the state of the scientific consensus on the effect of PA on cognition.

To date, there are two scientific consensus statements on the effect of physical activity and aging, and cognitive and academic performance [26,42]. Regarding aging, twenty-six researchers from nine different countries and a variety of academic disciplines met in Denmark to reach an evidence-based consensus about physical activity and its effect on older adults. According to the consensus statement, physical activity slows down age-associated cognitive decline and neurodegeneration in physically active adults. Additionally, acute moderate-intensity PA could produce short-term benefits in cognitive performance [42]. This consensus, however, was based on a face-to-face meeting. In such meetings, there is a conformity pressure to adjust one’s own opinion to that of the group, especially in homogeneous groups [43]. Because of this social pressure to conform with group norms, there is a risk that scientists may agree with the group consensus even though they may have different personal views.

### 1.2. Current Study

The goal of this consensus study was twofold: Firstly, to quantify the scientific consensus on whether physical activity has cognitive benefits. The present study distinguishes between physical activity and physical exercise, even though both terms are often used interchangeably. We further distinguish between acute and chronic effects of physical activity. Acute effects develop during short-term exposure or a single bout of physical activity, whereas repeated bouts of physical activity are needed for the chronic effects to take place [26]. Both effects are significant, with acute effects, on one hand, expressed as improved attention and cognitive function [13,26]. Chronic effects, on the other hand, are associated with brain structure changes, and improved learning and memory [13,26].

In the current review, physical activity is the field of interest. The present work aims to report on the actual consensus in the field, regardless of the form of physical activity and of the acute/chronic effects on cognition.

This goal was explored in the first stage of the study. During the first stage, a sample of scientific literature, published over a 15-year period, was examined to determine the level of scientific consensus in the field. Each abstract was categorized per author based on the level of endorsement (explicit endorsement, implicit endorsement, neutral, implicit rejection, explicit rejection, or partial endorsement/partial rejection). To prevent conformity biases, our study was anonymized so that scientists did not feel social pressure to conform to certain expectations, and thus, not refrain from expressing their own views.

The second goal was to explore if there is a gap between the general public’s and scientists’ interpretations of scientific texts. It was hypothesized that there would be no association between the scientists’ and laymen’s interpretations of scientific abstracts. This hypothesis was based on the discrepancy between the public’s and scientists’ views on key issues [44,45]. In the second phase, the same scientific literature was distributed to participants with a non-academic background. Each abstract was then classified by two independent raters, and if any disagreements arose, they were resolved by a third party; namely, an arbitrator. Upon completion of the final ratings, both scientists’ and non-scientists’ ratings were compared to see if there is an association between interpretations.

Nevertheless, our study differs from the approaches adopted by Singh et al. [27] and Bangsbo et al. [42] in three ways: First, we adopted a theory-based consensus approach, which differs from the expert panel approach by Singh et al. (2019) [27]. Instead of authors recommending international experts in the field of physical exercise, we contacted authors who published manuscripts related to the effects of PE on cognitive performance. This means that we contacted authors from a wide range of expertise. Secondly, our approach further investigated the degree of match-mismatch between understanding and beliefs of experts versus non-experts. Third, both experts and non-experts remained anonymous; hence, alleviating social conformity pressure.

Through analysis of physical activity-related manuscripts published from 2004 to 2019 by scientific and non-scientific participants, this study provides the first consensus analysis of its kind to quantify and evaluate the level of consensus in nearly two decades of research.

## 2. Materials and Methods 

### 2.1. Participants and Procedure

This study was approved by the Ethics Committee of the Department of Psychology, Education, and Child Studies of Erasmus Rotterdam University, the Netherlands (EUR/ESSB/DPECS/19052019). The method we used is similar to the one used by Cook et al. (2013) [46] for quantifying the consensus on anthropogenic global warming.

#### 2.1.1. First Stage: Endorsement from Self-ratings by Scientists

The participants in the first stage of the study were researchers. A researcher here can be defined as a person who carries out academic and/or scientific research. Therefore, people who had (co-) authored at least one theoretical/empirical paper or both in peer-reviewed journal articles were as researchers. They are interchangeably referred to as “scientists,” “authors” and “experts” throughout this paper. The email addresses of 744 scientists were collected, typically from the corresponding author and/or the first author on an article, while 729 scientists were contacted by using various social platforms. The authors were individually sent an invitation to participate in a survey in which they were asked to rate the abstracts of their own published manuscripts on PA and cognitive benefits. We could not reach 15 corresponding authors (2% drop-out rate) or their fellow researchers, either via email or via ResearchGate or other social media; i.e., Loop and LinkedIn.

**Figure 1:**
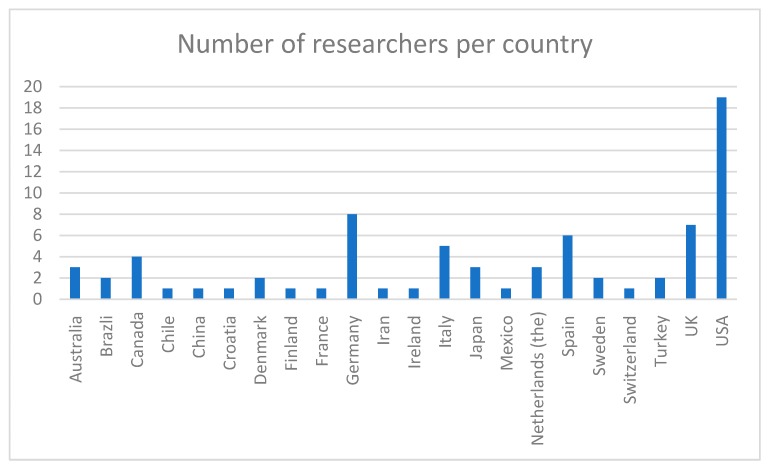
Numbers of researchers per country.

The same survey was distributed to all 729 authors via email. In the email, they received instructions in English and an explanation about the purpose of the current study. Additionally, they were directed to a list of all 729 scientists’ emails and their corresponding abstracts. Authors could easily identify which abstracts were theirs by entering their name in a keyword search in the list. The abstracts were rated by using a Likert scale with six answer options being “explicit endorsement,” “implicit endorsement,” “neutral,” “implicit rejection,” “explicit rejection,” and “partial endorsement/partial rejection.” Each category level was defined, and those definitions were distributed to scientists (see Table 1). The author with the most papers had 15 manuscripts, attributed to them by being the first and/or corresponding author. Those with the least amount had a single manuscript. Manuscripts’ abstracts were referred to as “questions” throughout the survey. The survey form was flexible, allowing researchers to answer for as many articles as were attributed to them by not making it mandatory to answer all 15 questions. Researchers received their own abstracts, so no randomization sequence was used. Reminder emails were sent to enhance response rates, but ultimately, the response rate was based on the judgment of the respondent. The first set of reminder emails was sent to all 729 scientists a week after the first email. A positive response was received from 75 researchers (10.3%). The sample consisted of 48 (64%) and 27 (36%) adult men and women, respectively. We could not report on the precise age of the scientists since such information is not available in journal articles or platforms such as ResearchGate. It can be obtained on LinkedIn; nonetheless, the majority of them did not own a LinkedIn account. The majority of scientists (see Figure 1) were from USA (25.3%, *n* = 19), Germany (10.6%, *n* = 8), the UK (9.3%, *n* = 7), and Spain (8%, *n* = 6).

#### 2.1.2. Second Stage: Endorsement from Abstract ratings by Non-Scientists

Recruitment was targeted at an English-speaking audience with no restriction of age, sex, and nationality by using social platforms such as Facebook and LinkedIn. Non-scientists who had obtained at least a C1 level certificate in English, as defined by the Common European Framework of Reference for Languages (CEFR; 2011) [47], were allowed to participate in the second stage of the study. Each volunteers’ language level was determined during individual correspondence. As intended, a total of 15 non-scientists (*n*_women_ = 13, 86.7%) were included (*Mean*_age_ = 23.26 years, *SD* age = 0.727, range 18–27 years). The non-scientists had diverse nationalities. Nationalities included Bulgarian (*n* = 8, 53.3%), Argentinian (*n* = 1, 6.7%), Brazilian (*n* = 1, 6.7%), Dutch (*n* = 1, 6.7%), Polish (*n* = 1, 6.7%), Portuguese (*n* = 1, 6.7%), and Swedish (*n* = 1, 6.7%). Three of the non-scientists had completed secondary education (20%) and 11 of them were either in a process of obtaining or had obtained a graduate diploma (73.3%). Only one volunteer had a postgraduate diploma (6.7%). Non-scientific participants were classified as young adults with no intellectual and developmental disabilities. The non-scientists were randomly assigned to five separate groups. Each group consisted of three people—two independent, anonymized raters and one arbitrator. The arbitrators resolved disagreements in category ratings. They did so by reading the abstracts and raters’ justifications on their category appraisal. The abstracts that were provided to the non-scientists were randomized and kept concealed until the group allocations were finalized. This sequence was held independently and remotely by the study leader. The difference between the survey used for the scientists and the one used for the non-scientists was in the abstract distribution. Scientists received their own abstracts, so no randomization sequence was used. The participants in the second stage were randomly distributed abstracts with a roughly equal representation of each category level per survey version (see Appendix A: Abstract Randomization). Abstracts were distributed to participants via a web-based system with only the title and the abstract being visible. Other information, such as authors’ names, affiliations, journals, and publishing dates, were hidden. The non-scientists filled in the surveys from their homes. No participant dropped out of the study.

### 2.2. Materials

#### 2.2.1. First Stage: Endorsement from Self-Ratings by Scientists

The survey consisted of the following sections: the first section contained instructions, informing scientists about the goal and the duration of the study. Participants were given the definitions of each endorsement category level in an email, and were further instructed to complete the survey in a single session. Initially, the survey used a 5-point Likert scale ranging from 1, “explicit endorsement” to 5 “explicit rejection”; however, one researcher marked that he/she could not place his/her article in either option. Therefore, the “partial endorsement/partial rejection” alternative was added for all researchers and one researcher was asked to fill in the survey once again, since he/she did not get the “partial endorsement/partial rejection” initially. Nonetheless, this option did not change the abstract categorizations for the researcher. Hence, the other scientists received a survey, containing abstract ratings, based on a 6-point Likert scale ranging from 1, “explicit endorsement” to 6 “Partial endorsement/partial rejection” (see Table 1). The scientists were assured that their data would be anonymous and that the personal information that they provided in the second section of the survey, i.e., personal email, would be solely used by the study leader to identify which researcher took part in the study. The third section dealt with the research category: It provided scientists with eight closed options and a ninth open option incase their research could not be placed in the former categories; namely, “neuroscience,” “sport sciences,” “clinical neurology,” “physiology,” “rehabilitation,” “psychology,” “education,” “cognitive science,” and “other.” The fourth section contained 15 closed-ended questions, using a 6-point Likert scale. For a detailed overview, see Appendix B. In the fifth section of the survey, scientists were asked for further comments or feedback regarding the study.

#### 2.2.2. Second Stage: Endorsement from Abstract ratings by Non-Scientists

Five different survey versions were distributed to 15 non-scientists. Each survey was based on the same template, each with a unique and randomized set of abstracts. Four of the surveys contained 31 abstracts and one had 32 abstracts. The survey was comprised of six sections. The first section contained instructions, informing participants about the goal and the duration of the study. Non-scientists were given the definitions of each category level (see Table 1) and were further instructed to complete the survey in a single session, using the same 6-point Likert scale ranging from 1, “explicit endorsement” to 6 “partial endorsement/partial rejection” as the “scientists.” Non-scientists were asked to rate what level of endorsement was reported in the abstract according to their judgement. The second section dealt with the collection of personal information. Sections three through five contained roughly nine to 12 abstracts each. Participants could take a break between sessions to prevent exhaustion. The sixth and last section contained an open question where non-scientists were asked if they had any comments or feedback regarding the study. For a brief overview of the survey, structure, and the question types, see Appendix C.

### 2.3. Systematic Search, Selection Protocol, and Final Sample of Manuscripts

**Figure 2:**
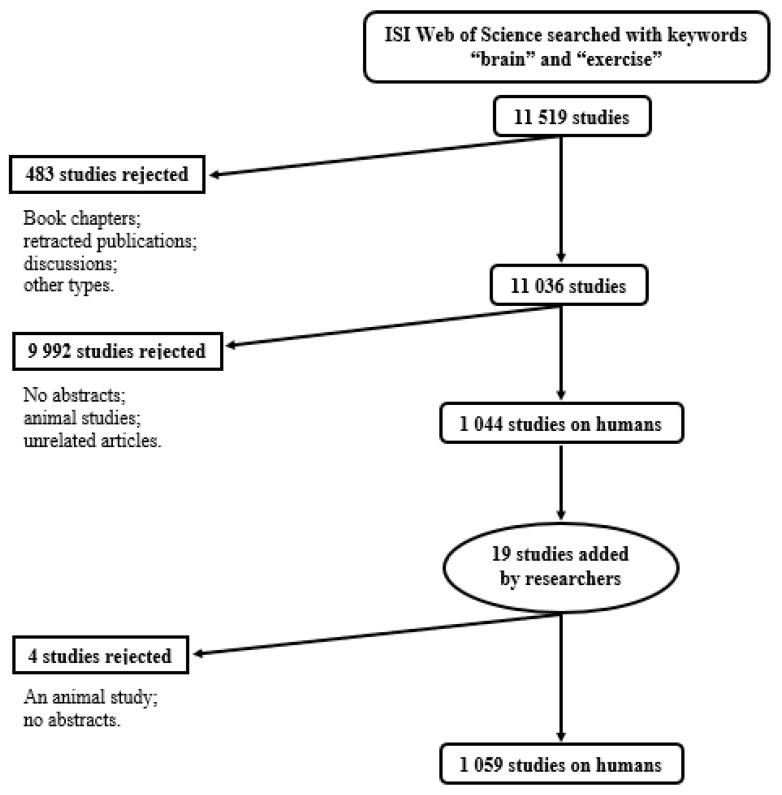
Flowchart of systematic search and selection protocol.

A search was conducted on September 2018 in ISI Web of Science with keywords “brain” and “exercise.” Although the aim of the study was to assess physical activity and cognition, those search terms (i.e, “physical activity” and “cognition”) did not produce relevant results. For instance, most of the studies in the database would measure physical activity but would fail to compare it to cognitive function. Therefore, even though it might appear that the search terms do not perfectly suit the aim of the study, they were used since they were broad enough to include relevant results with studies where cognitive function, brain structure, or brain function were assessed in relation to physical activity, not only exercise. Furthermore, Web of Science has an evaluation and selection process of content based on impact, influence, timeliness, and geographic representation, and allows only for peer-reviewed manuscripts. As such, our search was restricted to content complying with our search terms. The systematic search included mainly English, German, Spanish, and Russian language literature without any limitation of publication date. After removing duplicates and excluding book chapters, and grey literature, it was downsized to 11,036 manuscripts. Editorial materials, however, were included. According to a recent study, editorials qualify as a valuable extension to any bibliometric research and excluding them would be too “rigid” [48]. A flowchart of the systematic search and selection protocol can be found in Figure 2. The search yielded a result of 11,519 manuscripts.

The selected studies were transferred in Mendeley from where they were further reduced to 1044. First, manuscripts with no abstracts (*n* = 95) were excluded. Animal studies (*n* = 3141) were also removed since they are a poor predictor of human reactions [49].

Manuscripts that did not discuss cognitive changes were also removed (*n* = 6756). Overall, articles and editorials were included if human subjects were used or discussed. The inclusion criteria also included manuscripts from relevant academic domains such as psychology, sport sciences, and neuroscience.

In addition, during the first stage of the study, 19 manuscripts were added from the participating scientists in addition to the manuscripts they had been asked to rate, adding up to a total of 1063 manuscripts. These 19 studies were added spontaneously during the research, since answer options were not restricted. Some authors rated papers which were not listed under their names. They consequently disclosed their titles and abstracts so that they could be added to the study. The majority of these studies were unpublished manuscripts that were not part of the systematic search using the search engines in the current study. Such manuscripts were often from the “rejection” category and such input helped expand the literature included in the research. Only the manuscripts which met the inclusion criteria (articles and/or editorials, with abstracts, human subjects, and a relevant academic domain such as sport sciences) were included in the last stage of the research. Four studies were excluded, as they did not fit the inclusion criteria (i.e., one animal study and studies without abstracts; see Figure 2). Hence, the final number of manuscripts was 1059 (see Figure 2). Non-published work was added to minimize publication bias, since studies with significant results are mostly published [50].

The non-published studies were provided by scientists who participated in the research. The search was updated on November 2018 and no new studies were added from the updated search.

With the selected 1059 manuscripts, we started inviting the 729 scientists to participate in the study. As described earlier, 75 scientists answered the survey. Due to the low response rate, only 159 manuscripts were rated by their authors, and subsequently included in the current study.

## 3. Results

The academic field categorizations given by the scientists were analyzed. Most research manuscripts in the sample were in the fields of neurosciences (32.7%, *n* = 52), psychology (19.5%, *n* = 31), cognitive science (13.8%, *n* = 22), and sport sciences (13.2%, *n* = 21).

### 3.1. First Stage: Endorsement From Self-Ratings by Scientists

**Figure 3:**
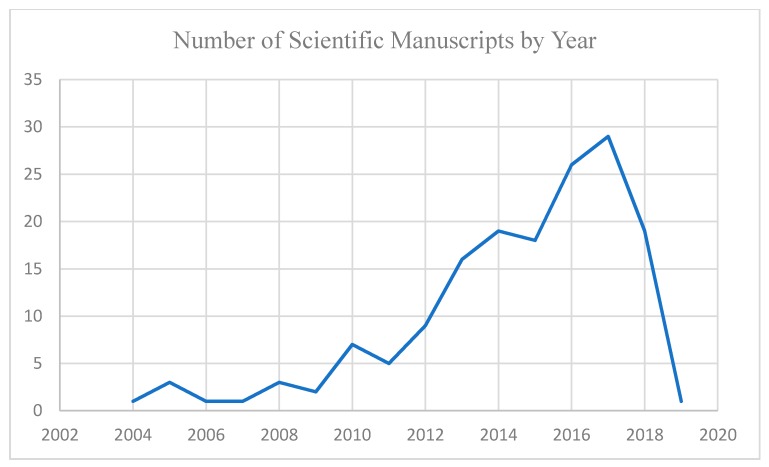
Number of scientific manuscripts by year.

The current research included 159 scientific manuscripts from 22 countries, published in the last 15 years (see Figure 3), and a total of 75 scientists and 15 non-scientists. Descriptive statistics were implemented, and no inferential statistical tests were conducted.

The self-rated levels of endorsement are shown in Table 2. Among respondents who authored a manuscript, 76.1% of the manuscripts endorsed the proposition that PA has a positive effect on cognitive outcome variables. Moreover, 80% of scientists endorsed the statement that physical activity improves cognition.

### 3.2. Second Stage: Endorsement from Abstract Ratings by Non-Scientists

Non-scientists’ abstract ratings, which are presented in Table 2, were consistent with scientists’ ratings.

### 3.3. Comparing the Results from the Scientists and Non-Scientists

A Pearson’s chi-squared test was conducted to check if there was a dependence between the answers which scientists had given and those given by the non-scientists. In this way, it could be checked if the manuscripts present in these surveys were perceived similarly by the two groups of participants-scientists and non-scientists. The analysis was performed in R studio at a 95% confidence interval and significance level 0.05. The resulting output showed statistically significant dependence, χ^2^ (5) = 15.91, *p* = 0.01. Hence, we found that in this survey scientists and non-scientists perceived the article summaries similarly in terms of the endorsement of the effect of PA on cognitive outcomes variables (see Table 3). The resulting χ^2^ coefficient, however, depends both on the dimension of the contingency table and on the size of the sample. Therefore, to eliminate the dependence on the sample size, the contingency coefficient was calculated to check for the strength of the χ^2^ test conducted as. The formula for the observed contingency coefficient was C_obs_ $=\frac{\sqrt{x^{2}}}{x^{2}+n}=0.30$. As coefficient C was still dependent on the dimension of the contingency table, it was normalized so that its range extended from 0.0 to 1.0 (C_corr_ = 0.42). The maximum of the contingency coefficient for this table was C_max_ = 0.81. The standardized contingency coefficient was C_stand_ = 0.52 which indicated that the relationship was strong between the variables; therefore, there was a statistically significant dependence between the scientists and non-scientists’ perceptions (standardized C = 0.52, *p* < 0.05).

## 4. Discussion

In the present study we tried to determine the scientific consensus regarding the effects of physical activity on cognition. Scientific consensus is a tool that serves as a form of social proof and hence, determining and quantifying the consensus is important for evidence-based policy. Science methodology and vocabulary are repeatedly deemed inaccessible to the general public, and they often do not specify what actions can be taken by individuals and organizations alike. The present study was an attempt to help make the first step to addressing these challenges by communicating the scientific consensus and helping to realign public views with experts’ opinions.

Overall, the first stage of the study corresponded to a previous consensus statement, released by Bangsbo et al. (2019) [42]. In particular, scientists (80%) reached a consensus that physical activity benefited cognitive processes and brain health based on the surveyed literature (76.1%), similar to a previous consensus on aerobic physical activity and cognitive function in older adults [42]. The scientists’ ratings were similar to those of the non-scientists’ (70.4%). Despite this, however, there was a methodological difference between the study of Bangsbo and colleagues and the current study. Unlike Bangsbo et al. (2019) [42], where scientists had a face-to-face meeting, the present study required the respondents to remain anonymous. This was done to eliminate bias and peer pressure that could occur in face-to-face meetings. When considering the second stage of the review, non-scientists were able to interpret scientific abstracts in the way scientists intended (standardized C = 0.52, *p* < 0.05). The results were surprising, since it was expected that there would be no association between the scientists’ and laymen’s interpretations of scientific abstracts. A disadvantage of the contingency coefficient, however, is that its maximum possible value depends on the number of cells in the table. Therefore, for future replication studies, it is advisable to adhere to the same number of cells in the table to allow for comparability across studies.

As a further factor of consideration, the database search was set to include all published manuscripts from 1913 until 2018, covering 105 years of scientific inquiry. Due to the selection protocol, the 1060 manuscripts selected were published between 1991 and 2018; thus, covering 27 years of research. They were from peer-reviewed journals only. Unfortunately, the current review covered a much smaller span, representing 15 years of research due to the low response rate (10.3%). For future studies that manage to include earlier research in their study, it could be interesting to conduct a time series analysis of each level of endorsement, analyzed in terms of the number and the percentage of abstracts to see if consensus changed over the years, and if so, how.

Overall, we found a 76.1% consensus that physical activity has cognitive benefits. In fact, experts’ and laymen’s abstract ratings were similar, which means that non-scientists could correctly understand and interpret the scientific language used in abstracts. This is important, since as mentioned earlier, communicating the scientific consensus is a powerful tool for assessing the weight of scientific evidence and for realigning public views of the issue with expert opinions [35]. However, it should be noted that different disciplines produce different answers. To illustrate, 75% (*n* = 9) of the rejection endorsing studies were in the field of neuroscience. The other three rejection endorsement manuscripts are in the fields of sport sciences (17%, *n* = 2) and rehabilitation (8%, *n* = 1). Therefore, in our sample, there were no rejection endorsing manuscripts from research in psychology, clinical neurology, or cognitive science. This could be attributed to the differences in methodology and cognitive performance measurement methods. A general problem in the methodology of exercise research is the relative lack of single standardized criteria for defining “exercise” and determining its effectiveness, so more standardized definitions would be useful in future research [51]. It is also interesting to note that the criteria for establishing cognitive impairment were not consistent across studies, making it difficult to compare the differential effects of exercise across diagnostic categories (such as neurodevelopmental disorders and anxiety disorders), concluding that more standardized criteria are needed. Adhering to official guidelines, such as World Health Organization’s guidelines, on definitions of PE and PA and on the recommended intensity, duration, and volume of PA per age group, could be effective for comparative research. Further research is required from a range of disciplines to advance the understanding of the theoretical models of cognitive enhancement. For instance, forms of physical activity, such as balance and resistance training, have been insufficiently explored. More evidence is needed on the cognitive effects of such forms of PA, specifically in older adults [42].

Such interventions could also be designed to be executed in a straightforward manner, and preferably, in real-life tasks. Likewise, cognitive research has expressed a disproportionate interest in the effects of PA on aspects, such as executive function and working memory. Research within the child and developmental studies have pointed out the importance of learning processes, induced by motor movements [25]. Nonetheless, there is a scarceness of research on the effects of learning outside of the field of child and developmental studies. More systematic incorporation of various movements such as gesturing, head-tilting, and tracing movements could contribute to the ability to design and implement ecologically valid interventions that combine complex and diverse environments with efficacy for both cognitive and neural health. Concomitantly, a comprehensive battery of neuropsychological assessments and neuroimaging tools would complement behavioral findings [52]. Future studies would benefit from such measures since they would significantly contribute to the understanding of how PA-induced changes in neural circuitry might be associated with accompanying behavioral changes [52].

In the age of globalization, social and life sciences have remained largely American and European. An empirical review study has noted that volunteers from Western, educated, industrialized, rich, and democratic countries are the usual spokesmen of humanity who think differently than those from other parts of the world [53]. Therefore, there is a need for replication studies on the effects of a physical activity intervention on cognition with participants from different nationalities. Additionally, another study has indicated the difficulty of estimating the true robustness and effect size of the cognitive enhancement effect by surveying the published literature [54]. Publication bias has been implicated in decreasing the efficiency of the science and bringing the credibility of published research to lower standards [54]. Hence, replication studies are needed to combat the publication bias and low diversity in the scientific literature. To conclude, remarkable progress has been made in the scientific understanding of the PA–cognition correlation during normal development, as well as in psychiatric and neurological populations. Studies have provided promising support for PA to be associated with both early and late-life cognitive functioning.

### 4.1. Study Limitations

There are several limitations in the present literature analysis, such as the representativeness of the literature sample, lack of clarity in the abstracts, the scholars’ sample representativeness and risk of evaluation bias.

The issue of sample representativeness when investigating extensive concepts, such as PA and cognition, was addressed by selecting a large sample for this type of literature analysis by using broad search terms (i.e., “brain” and “exercise”). Nonetheless, 1060 manuscripts are still a small fraction (9.2%) of the physical activity and cognition literature, since a Web of Science search yielded 11,519 manuscripts. Additionally, the search terms we used were a possible limitation. Therefore, the sourcing techniques employed in the current analysis could be expanded to include more manuscripts by using multiple databases, such as Scopus, Cochrane, PubMed, PsycINFO, and many others. Additionally, a combination of different search terms, including “physical activity, exercise, aerobic exercise,” and “cognition, cognitive function, executive function, learning, memory, academic, cognitive, and learning performance” could be more informative for future research.

Another area of uncertainty is the nature of the language used for writing the abstracts and abstract formats. In some cases, the ambiguous language made it difficult for the second stage laymen to determine the intended meaning of the authors. The implementation of the authors’ self-rating process allowed us to compare their abstract categorizations to those of laymen. The descriptive analysis revealed that, whereas non-scientists categorized nine papers as belonging to the uncertain category due to ambiguous language and dissimilar abstract formats, scientists categorized only one paper as belonging to the uncertain category. With regard to the “scholars sample representativeness,” it may be questionable whether someone with one published paper in the field should be considered a representative of the field. This, in turn, would have lowered the reliability of the survey and decreased response and completion rates [55].

Possibly, the characteristics and the size of the non-expert group may not have been large enough to make a sensible comparison with the expert group. We deliberately opted for non-representativeness in our study design (“intentional” non-representativeness) for a couple of reasons. The first reason is practical; recruiting a large number of volunteers is challenging and not always feasible. Additionally, if we had matched the non-expert sample size to that of experts, the number of manuscripts per volunteer would have decreased to approximately two manuscripts per a non-expert. This means that the survey would not be representative of the different categories of endorsement, and it could have increased the content validity bias. Secondly, we aimed at the age group of 15–64, as it represents 65% of the current world population [56]. By choosing this age group for the non-expert group in our study, the findings were more representative on a global scale. However, it should be noted that these findings should not be extrapolated to other non-expert age groups. Furthermore, the non-expert group cannot be considered as a representative sample due to its small size and the selection protocol employed. Future research should increase the size of the non-expert group relative to the one of experts’ if the manuscript sample is big enough to allow for sufficient representativeness of the different categories of endorsement.

What is more, participants’ subjectivity is inherent in the abstract rating process for both experts and non-experts. Regardless of defining the criteria for determining ratings prior to the rating period, it is possible that experts showed evaluation bias. Such a source of rating bias is that experts might have been more likely to classify papers as sharing the endorsement if they themselves were endorsing it, regardless of what the paper says. Another source of subjectivity bias could be scientific reticence which would mean that scientists would be more biased towards a “no position” categorization to avoid conflict. This bias was partially addressed by using multiple independent raters and comparing the abstract categorization results to those of author self-ratings. A comparison between both types of ratings revealed that this bias had minimal impact on the level of consensus. Authors’ “no position” categorizations (*n* = 26, 16.4%) were almost identical to those of volunteers’ (*n* = 25, 15.7%).

### 4.2. Government Policies

Scientists often aspire to see their findings being used to benefit society. Consecutively, decision-makers such as civic organizations, government officials, and citizens often seek out the best scientific evidence to make well-informed decisions for policies and regulations [35]. The scientific evidence can be used to improve practice, especially when research findings are results of well-designed studies with methodological rigor that minimized the chances of bias. For instance, international public health agencies such as the World Health Organization often implement research findings into their policies, which serves to highlight the importance of research findings in daily life [57]. The findings from the current study could contribute to efforts to translate research evidence into effective community programs for older adults with and without cognitive impairments, and among children and young people.

## 5. Conclusions

The purpose of this review was to determine the scientific consensus on the cognitive effects of physical activity. There was a 76.1% consensus that physical activity has positive effects on cognition in humans, corresponding to a previous consensus, released by 25 scientists [42]. The present consensus was drawn from categorizations, made by 75 scientists which makes it the biggest consensus to date. In categorizing the studies with respect to their level of endorsement, it became clear that the general public’s interpretations of scientific texts did not differ from the scientists’ interpretations. This finding suggests that the public can guard itself against influential misinformation by assessing the scientific weight of evidence themselves, instead of relying on mass media. A future follow-up investigation is required to expand the current analysis by incorporating a sourcing technique that includes a greater number of active scientists in the field and their corresponding research.

## Figures and Tables

**Table 1:** Definitions of each level of categorization.

Level of Endorsement	Description	Example
1. Explicit endorsement	Clearly states that physical activity improves cognition	“… combined muscle strengthening and aerobic conditioning were able to improve cognitive performance and increase BDNF ^a^”
2. Implicit endorsement	States that physical	“Providing 45 min of daily
	activity improves cognition or refers to it as a known fact	physical activity can perhaps increase cognitive ability…”
3. Neutral	Does not address or mention any correlation/causation between physical activity and cognition	“… physical activity within 7 days of acute injury compared with no physical activity was associated with reduced risk of PPCS ^b^ at 28 days”
4. Implicit rejection	Minimizes or rejects that there is a positive influence on cognition	“Those associations were… non-significant after controlling for age and Expanded Disability Status Scale scores”
5. Explicit rejection	Explicitly rejects that physical activity has a positive influence on cognition	“An 8-week physical activity intervention… neither benefits cognitive function nor affects the levels of the serum proteins analysed in nonagenarians”
6. Partial endorsement/Partial rejection	The results support the notion that physical activity benefits cognition; however, it also partially rejects it	“For the more cognitively demanding stimuli, physical activity was positively related to the linear increase in accuracy… and inversely related to the quadratic decelaration of accuracy gains”

Note. ^a^ BDNF = brain-derived neurotrophic factor. ^b^ PPCS = persistent postconcussive symptoms.

**Table 2:** Self-ratings for each level of endorsement (percentages and total number of manuscripts for scientists and non-scientists).

Position	Percentage of all Abstracts for Scientists		Percentage of All Scientists	Percentage of All Abstracts for Non-Scientists	
Endorse	76.1%	(*n* = 121)	80.0% (*n* = 60)	70.4%	(*n* = 112)
Explicit	52.2%	(*n* = 83)	53.3% (*n* = 40)	37.1%	(*n* = 59)
Implicit	23.9%	(*n* = 38)	26.6% (*n* = 20)	33.3%	(*n* = 53)
No position	16.4%	(*n* = 26)	13.3% (*n* = 10)	15.7%	(*n* = 25)
Reject	6.9%	(*n* = 11)	5.3% (*n* = 4)	8.2%	(*n* = 13)
Explicit	1.9%	(*n* = 3)	2.6% (*n* = 2)	5.0%	(*n* = 8)
Implicit	5.0%	(*n* = 8)	2.6% (*n* = 2)	3.2%	(*n* = 5)
Uncertain	0.6%	(*n* = 1)	1.3% (*n* = 1)	5.7%	(*n* = 9)

**Table 3:** Comparison of non-scientists’ abstract rating to self-ratings.

Position	Scientists	Non-Scientists
Endorse	76.1% (*n* = 121)	70.4% (*n* = 112)
No position	16.4% (*n* = 26)	15.7% (*n* = 25)
Reject	6.9% (*n* = 11)	8.2% (*n* = 13)
Uncertain	0.6% (*n* = 1)	5.6% (*n* = 9)

## Appendix A

**Table A1:** Abstract Randomization.

Category level	Version 1	Version 2	Version 3	Version 4	Version 5
Explicit endorsement	17	16	17	15	17
Implicit endorsement	7	8	7	8	8
Neutral	5	5	5	6	5
Implicit rejection	1	2	2	2	2
Explicit rejection	1	1	1	0	0
Partial endorsement/partial rejection	1	0	0	0	0

## Appendix B

**Table A2:** Survey Structure and Question Types for the First Stage of the Study.

No.	Section	Number of Questions	Survey Question Types
1	Instructions	-	-
2	Personal	1	Open-ended question (short-answer text)
	information		
3	Research	1	Close-ended questions (multiple-choice options with one open
	category		option if researcher’s field is not mentioned)
4	Abstract	1–15	6-point Likert scale from explicit endorsement to partial
	categorization		endorsement/partial rejection).
5	Feedback	-	Open-ended question (short-answer text)

## Appendix C

**Table A3:** Survey Structure and Question Types for the Second Stage of the Study.

No.	Section	Number of Questions	Survey Question Types
1	Instructions	-	-
2	Personal	1	Open-ended question (short-answer text)
	information		
3	Abstract	9–12	Close-ended questions (using a 6-point Likert scale from
	categorization		explicit endorsement to partial endorsement/partial rejection)
4	Abstract	9–12	Close-ended questions (using a 6-point Likert scale from
	categorization		explicit endorsement to partial endorsement/partial rejection)
5	Abstract	9–12	Close-ended questions (using a 6-point Likert scale from
	categorization		explicit endorsement to partial endorsement/partial rejection)
6	Feedback	-	Open-ended question (short-answer text)

## References

[1] International Health, Racquet & Sportsclub Association (2018). The 2018 IHRSA Global Report. The State of the Health Club Industry.

[2] Chau J., Reyes-Marcelino G., Burnett A., Bauman A., Freeman B. (2018). Hyping health effects: A news analysis of the ‘new smoking’ and the role of sitting. Br. J. Sports Med..

[3] Vallance J., Gardiner P., Lynch B., D’Silva A., Boyle T., Taylor L., Johnson S.T., Buman M.P., Owen N. (2018). Evaluating the evidence on sitting, smoking, and health: Is sitting really the new smoking?. Am. J. Public Health.

[4] World Health Organization (2010). Global Recommendations on Physical Activity for Health.

[5] U.S. Department of Health and Human Services (2018). Physical Activity Guidelines for Americans.

[6] Campbell N., De Jesus S., Prapavessis H., Gellman M.D., Turner J.R. (2013). Physical Fitness. Encyclopedia of Behavioral Medicine.

[7] Donnelly J.E., Hillman C.H., Castelli D., Etnier J.L., Lee S., Tomporowski P., Lambourne K., Szabo-Reed A.N. (2016). Physical activity, fitness, cognitive function, and academic achievement in children: A systematic review. Med. Sci. Sports Exerc..

[8] Dresler M., Sandberg A., Ohla K., Bublitz C., Trenado C., Mroczko-Wąsowicz A., Kühn S., Repantis D. (2013). Non-pharmacological cognitive enhancement. Neuropharmacology.

[9] Sibley B., Etnier J. (2003). The relationship between physical activity and cognition in children: A meta-analysis. Pediatr. Exerc. Sci..

[10] Hamer M., Chida Y. (2009). Physical activity and risk of neurodegenerative disease: A systematic review of prospective evidence. Psychol. Med..

[11] Vogt T., Schneider S., Anneken V., Strüder H. (2013). Moderate cycling exercise enhances neurocognitive processing in adolescents with intellectual and developmental disabilities. Res. Dev. Disabil..

[12] Smith P., Blumenthal J., Hoffman B., Cooper H., Strauman T., Welsh-Bohmer K., Browndyke J.N., Sherwood A. (2010). Aerobic exercise and neurocognitive performance: A meta-analytic review of randomized controlled trials. Psychosom. Med..

[13] Mandolesi L., Polverino A., Montuori S., Foti F., Ferraioli G., Sorrentino P., Sorrentino G. (2018). Effects of physical exercise on cognitive functioning and wellbeing: Biological and psychological benefits. Front. Psychol..

[14] Erickson K., Raji C., Lopez O., Becker J., Rosano C., Newman A., Gach H.M., Thompson P.M., Ho A.J., Kuller L. (2010). Physical activity predicts gray matter volume in late adulthood: The cardiovascular health study. Neurology.

[15] Brinke L., Bolandzadeh N., Nagamatsu L., Hsu C., Davis J., Miran-Khan K., Liu-Ambrose T. (2014). Aerobic exercise increases hippocampal volume in older women with probable mild cognitive impairment: A 6-month randomised controlled trial. Br. J. Sports Med..

[16] Tremblay S., Pascual-Leone A., Théoret H. (2018). A review of the effects of physical activity and sports concussion on brain function and anatomy. Int. J. Psychophys..

[17] Brisswalter J., Collardeau M., René A. (2002). Effects of acute physical exercise characteristics on cognitive performance. Sports Med..

[18] Mata J., Thompson R., Gotlib I. (2010). BDNF genotype moderates the relation between physical activity and depressive symptoms. Health Psychol..

[19] Gujral S., Manuck S., Ferrell R., Flory J., Erickson K. (2014). The BDNF Val66Met polymorphism does not moderate the effect of self-reported physical activity on depressive symptoms in midlife. Psychiatry Res..

[20] Marmeleira J. (2012). An examination of the mechanisms underlying the effects of physical activity on brain and cognition. Eur. Rev. Aging Phys. Act..

[21] Raji C.A., Merrill D.A., Eyre H., Mallam S., Torosyan N., Erickson K.I., Lopez O.L., Becker J.T., Carmichael O.T., Gach H.M. (2016). Longitudinal relationships between caloric expenditure and gray matter in the cardiovascular health study. J. Alzheimer’s Dis..

[22] Paas F., Sweller J. (2012). An evolutionary upgrade of cognitive load theory: Using the human motor system and collaboration to support the learning of complex cognitive tasks. Educ. Psychol. Rev..

[23] Sweller J., van Merriënboer J., Paas F. (2019). Cognitive architecture and instructional design: 20 years later. Educ. Psychol. Rev..

[24] Risko E., Gilbert S. (2016). Cognitive offloading. Trends Cogn. Sci..

[25] Sepp S., Howard S., Tindall-Ford S., Agostinho S., Paas F. (2019). Cognitive load theory and human movement: Towards an integrated model of working memory. Educ. Psychol. Rev..

[26] Mavilidi M., Ruiter M., Schmidt M., Okely A., Loyens S., Chandler P., Paas F. (2018). A narrative review of school-based physical activity for enhancing cognition and learning: The importance of relevancy and integration. Front. Psychol..

[27] Singh A.S., Saliasi E., Van Den Berg V., Uijtdewilligen L., De Groot R.H., Jolles J., Andersen L.B., Bailey R., Chang Y.K., Diamond A. (2019). Effects of physical activity interventions on cognitive and academic performance in children and adolescents: A novel combination of a systematic review and recommendations from an expert panel. Br. J. Sports Med..

[28] Lytle M., Bilt J., Pandav R., Dodge H., Ganguli M. (2004). Exercise level and cognitive decline. Alzheimer Dis. Assoc. Disord..

[29] Li G., Shen Y., Chen C., Zhau Y., Li S., Lu M. (1991). A three-year follow-up study of age-related dementia in an urban area of Beijing. Acta Psychiatr. Scand..

[30] Yoshitake T., Kiyohara Y., Kato I., Ohmura T., Iwamoto H., Nakayama K., Ohmori S., Nomiyama K., Kawano H., Ueda K. (1995). Incidence and risk factors of vascular dementia and Alzheimer’s disease in a defined elderly Japanese population: The Hisayama study. Neurology.

[31] Brasure M., Desai P., Davila H., Nelson V., Calvert C., Jutkowitz E., Butler M., Fink H.A., Ratner E., Hemmy L.S. (2018). Physical activity interventions in preventing cognitive decline and Alzheimer-type dementia. Ann. Intern. Med..

[32] Time. http://time.com.

[33] STAT. https://www.statnews.com.

[34] Tversky A., Kahneman D. (1971). Availability: A heuristic for judging frequency and probability. Psycextra Dataset.

[35] Maibach E., Linden S. (2016). The importance of assessing and communicating scientific consensus. Environ. Res. Lett..

[36] Graham B., Regehr G., Wright J.G. (2003). Delphi as a method to establish consensus for diagnostic criteria. J. Clin. Epidemiol..

[37] Landeta J. (2006). Current validity of the Delphi method in social sciences. Technol. Forecast. Soc. Chang..

[38] Cognitive Training Data. www.cognitivetrainingdata.org.

[39] Max Planck Institute for Human Development and Stanford Center on Longevity. http://longevity.stanford.edu.

[40] Simons D., Boot W., Charness N., Gathercole S., Chabris C., Hambrick D., Stine-Morrow E. (2016). Do “brain-training” programs work?. Psychol. Sci. Public Interest.

[41] Palmatier R., Houston M., Hulland J. (2017). Review articles: Purpose, process, and structure. J. Acad. Mark. Sci..

[42] Bangsbo J., Blackwell J., Boraxbekk C., Caserotti P., Dela F., Evans A.B., Jespersen A.P., Gliemann L., Kramer A.F., Lundbye-Jensen J. (2019). Copenhagen Consensus statement 2019: Physical activity and ageing. Br. J. Sports Med..

[43] Schulz-Hardt S., Frey D., Lüthgens C., Moscovici S. (2000). Biased information search in group decision making. J. Personal. Soc. Psychol..

[44] Hunter P. (2016). The communications gap between scientists and public. More scientists and their institutions feel a need to communicate the results and nature of research with the public. EMBO Rep..

[45] McFadden B. (2016). Examining the gap between science and public opinion about genetically modified food and global warming. PLoS ONE.

[46] Cook J., Nuccitelli D., Green S.A., Richardson M., Winkler B., Painting R., Way R., Jacobs P., Skuce A. (2013). Quantifying the consensus on anthropogenic global warming in the scientific literature. Environ. Res. Lett..

[47] Council of Europe (2011). Common European Framework of Reference for Languages: Learning, Teaching, Assessment.

[48] Van Leeuwen T., Costas R., Calero-Medina C., Visser M. (2012). The role of editorial material in bibliometric research performance assessments. Scientometrics.

[49] Bracken M. (2009). Why animal studies are often poor predictors of human reactions to exposure. J. R. Soc. Med..

[50] Hubbard R., Armstrong J. (1997). Publication bias against null results. Psychol. Rep..

[51] Colcombe S., Kramer A.F. (2003). Fitness effects on the cognitive function of older adults. Psychol. Sci..

[52] Prakash R., Voss M., Erickson K., Kramer A. (2014). Physical activity and cognitive vitality. Annu. Rev. Psychol..

[53] Henrich J., Heine S.J., Norenzayan A. (2010). The weirdest people in the world?. Behav. Brain Sci..

[54] Ingre M., Nilsonne G. (2018). Estimating statistical power, posterior probability and publication bias of psychological research using the observed replication rate. R. Soc. Open Sci..

[55] Kost R.G., de Rosa J.C. (2018). Impact of survey length and compensation on validity, reliability, and sample characteristics for Ultrashort-, Short-, and Long-Research Participant Perception Surveys. J. Clin. Transl. Sci..

[56] United Nations Population Fund. https://www.unfpa.org/data/world-population-dashboard.

[57] World Health Organization (2019). Risk Reduction of Cognitive Decline and Dementia.

